# Remineralization of Early Enamel Lesions with Apatite-Forming Salt

**DOI:** 10.3390/dj11080182

**Published:** 2023-07-28

**Authors:** Clifton M. Carey

**Affiliations:** School of Dental Medicine, University of Colorado, Aurora, CO 80045, USA; clifton.carey@cuanschutz.edu; Tel.: +1-303-724-1046

**Keywords:** remineralization, early enamel lesion, calcium phosphate, rinse, SalivaMAX

## Abstract

Objectives: This study sought to evaluate the remineralization of ex vivo human teeth using commercially available artificial saliva, SalivaMAX^®^, a supersaturated calcium phosphate rinse (SSCPR). Methods: early enamel lesions were artificially induced on ex vivo human teeth by chemical means. The teeth were exposed to the SSCPR for two minutes (experimental) or dH_2_O (control) four times per day for a total of 35 days. At time points of 0, 2.5, 21, and 35 days, micro-CT was utilized to determine the mineral density profile across the lesion and evaluate lesion depth. The relative percent remineralization was calculated from the initial lesion depth (Time 0) at each evaluation time. Student’s t-test was used to compare the extent of remineralization between the SSCPR and control groups for statistical significance at each time. To evaluate the changes in percent remineralization over time, a two-way ANOVA was used. Results: At Time 0 and 2.5 days, there was no difference in the percent remineralization between the SSCPR and control groups (*p* > 0.05). After 21 days, the teeth exposed to the SSCPR remineralized 56.7 ± 3.7%, while the control only remineralized 10.7 ± 11.0% (*p* < 0.0001). At day 35, the remineralization was 73.7 ± 5.4% and 18.2 ± 10.8% (*p* < 0.0001) for the SSCPR and control groups, respectively. Conclusions: A marked increase in remineralization occurred with the use of the SSCPR. Notably, the remineralization of the SSCPR occurred deep within the tooth and progressed toward the surface over time.

## 1. Introduction

**Figure 1:**
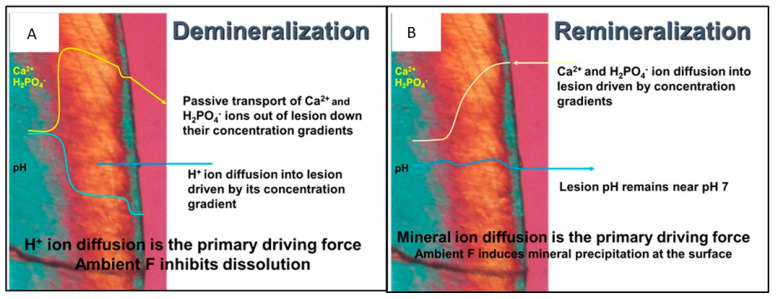
A polarized light micrograph of a white spot lesion with the mechanisms for demineralization (**A**) where an acid attack causes dissolution of enamel mineral at the lesion boundary followed by passive diffusion of calcium and phosphate ions out of the tooth, and (**B**) the mechanism for remineralization where calcium and phosphate ions diffuse from a higher concentration outside of the tooth into the WSL where precipitation occurs on existing apatite mineral at the lesion boundary progressing to the enamel surface.

Early enamel lesions are the earliest signs of enamel demineralization which, if left untreated, can develop into white spot lesions, cavitations, and tooth loss [1]. Microbial plaque is the primary etiologic agent of dental caries and the formation of white spot lesions [2]. Plaque bacteria utilize carbohydrates for energy, creating an acid byproduct. This acid byproduct causes a drop in the pH of the plaque fluid adjacent to the enamel, which induces enamel breakdown, dissolution, and decay [3,4]. One caries risk factor is xerostomia, also called dry mouth [5]. This is because saliva buffers the acid produced during demineralization and also provides calcium to the tooth, resulting in remineralization. This de/remineralization cycle results in a steady state where no net tooth loss occurs. When the saliva is not available, the remineralization phase is not sufficient to maintain the tooth, and net demineralization occurs. Figure 1 shows a polarized light micrograph of a WSL in a cross-section, demonstrating the mechanism of enamel demineralization and remineralization. Figure 1a shows the WSL under acidic attack: H^+^ ions (acid) diffuse into the enamel, dissolving the most soluble salts within the enamel, such as carbonate apatites and non-stoichiometric apatite crystals. The dissolution front (lesion boundary) is the zone of enamel loss between the sound enamel (blue) and the body of the WSL (orange). The process of demineralization essentially purifies the enamel mineral by dissolving the most soluble of the apatitic salts in the enamel. Thus, the lesion boundary is the advancing front of the lesion and the lesion advances from the deepest zone, creating high local concentrations of calcium and phosphate ions, which diffuse away from the advancing front. The dissolved calcium and phosphate ions passively move down a concentration gradient to diffuse out of the lesion and are lost from the tooth. As the diffusing calcium and phosphate ions reach the surface of the tooth, they may bind with available fluoride at the surface and precipitate forming a mineral-dense intact layer at the surface of the tooth, which is blue in Figure 1. Note that the outside of the tooth is the purple zone of the image. The loss of calcium is the rate-limiting step for the advancement of the lesion [6]. 

Remineralization has been defined by Cochrane et al. “… as the process whereby calcium and phosphate ions are supplied from a source external to the tooth to promote ion deposition into crystal voids in demineralized enamel, to produce net mineral gain [7].” It is the deposition of new minerals (increasing mineral density) within a lesion. It requires calcium phosphate ions to be reintroduced into the lesion where they can precipitate. Figure 1b shows the mechanism for remineralization of a WSL. Concentrations of calcium and phosphate ions greater than the concentrations in the lesion are needed to remineralize a lesion. Calcium and phosphate ions diffuse into the enamel through micropores of the intact layer and inter-crystalline voids of the lesion. While the concentration of the calcium and phosphate ions must be high enough to diffuse into the tooth, the concentration must not be high enough to precipitate at the surface plugging tubules and preventing calcium and phosphate ions to enter the WSL. Saliva contains calcium and phosphate ions at concentrations that result in supersaturation with respect to hydroxyapatite [8]. Therefore, under conditions where the caries challenge has been removed, saliva is often able to slowly remineralize early lesions [9]. The calcium and phosphate concentrations augment the slightly higher calcium and phosphate concentration at the lesion boundary to precipitate, eventually remineralizing the whole lesion. The most effective remineralization should occur at the lesion boundary and proceed toward the surface of the tooth.

Remineralization and prevention of early enamel lesions and WSLs are often conflated by dental researchers and professionals. There are many studies of dental products that report the prevention of dental caries associated with topical fluoride or calcium phosphate therapies [10]. Some of those claim that caries prevention is remineralization. The problem is that the remineralization of an existing lesion is not the same as the deposition of mineral ions during an acidic cycle where these act as a buffer that protects the tooth mineral. This lack of precision within the dental literature is unfortunate because manufacturers and practitioners have exploited this imprecision. Fortunately, the U.S. Food and Drug Administration (FDA) has policed the manufacturers’ advertising claims and required those who have promoted their products as remineralizing or have the ability to replace lost minerals within a lesion to provide clinical evidence or withdraw the advertising. At present, the FDA does not allow a remineralizing claim for any dental product. While there are in vitro studies that demonstrate actual remineralization of an existing lesion, high-quality clinical studies are needed to show clinical efficacy.

Efforts to reverse (remineralize) WSLs once they are formed focus on changing the environment at the WSL such that the acid-forming dental plaque is disrupted and on providing higher concentrations of calcium and phosphate mineral ions such that the mineral ions can be taken up by the porous WSL, depositing new mineral within the WSL when the mineral ions precipitate. Several approaches to achieve remineralization have been suggested [10] including bioactive peptides to help guide mineral formation in the presence of high concentrations of calcium and phosphate ions [11] and applications of protein-stabilized concentrations of calcium phosphate complexes (casein phosphopeptide amorphous calcium phosphate, CPP-ACP), which release calcium and phosphate to the tooth during cyclic acid production of the plaque bacteria only when plaque pH becomes acidic at pH 5 to 5.5 [12]. Another common approach has been to apply a fluoride varnish to the WSL where the deposited fluoride serves to attract calcium ions to the lesion [13]. A recent review by Fernandez-Ferrer et al. [14] concluded that none of the treatments (CPP-ACP paste or fluoride varnish) were capable of remineralizing WSLs and that more studies are needed. In a randomized controlled trial to evaluate the effectiveness of two different fluoride products (CPP-ACP paste plus and fluoride varnish) compared to usual at-home oral care with fluoridated toothpaste, it was reported that neither the CPP-ACP paste nor the fluoride varnish was more effective than normal at-home hygiene with fluoridated toothpaste to improve the appearance of WSLs over 8 weeks after the removal of orthodontic appliances [15].

While there are no other studies that report the remineralization of early enamel lesions using the SSCPR product, there have been several studies on WSL remineralization. One such study was reported by Cochran et al. [12] on the ability of casein phosphopeptide stabilized amorphous calcium phosphate (CPP-ACP) and amorphous calcium fluoride phosphate (CPP-ACFP) to remineralize enamel subsurface lesions. The CPP binds free calcium, fluoride, and phosphate ions such that the complexes do not precipitate mineral ions at neutral pH. This stabilization of calcium phosphate is reduced when the pH is lowered to pH 5.5 and below, releasing the mineral ions. At a low pH, subsequent precipitation of apatite-like compounds can then occur. Unless the pH is lowered, the mineral ions are not released from the CPP complexes, but rather only at a pH that is at or below the critical pH where the enamel is at risk of dissolution. 

The use of nano-hydroxyapatites (nHAp) has been under investigation for several decades. The logic is that nHAp diffuses into and lodges within the lesion, which later is dissolved and integrated with the mineral of the tooth when there is an acidic cycle [16]. However, the efficacy of nHAp-containing dental products for lesion remineralization is controversial. There is in vitro and clinical evidence that nHAp may remineralize artificial early lesions better than fluoride varnish (5% NaF). Najibfard et al. [17], on the basis of a non-controlled crossover in situ study, reported that dentifrices containing either 5% or 10% nHAp caused 14 to 10% remineralization of artificial lesions (respectively), which was comparable to an 1100 ppm NaF dentifrice after 28 days. It is unclear what the remineralizing effect of the saliva alone would have produced in this study. However, a recent review declared that at this time, the evidence is insufficient to support conclusions about the effect of nHAp on the remineralization of early lesions [18].

A recent review by Singal et al. [19] on the topic of calcium phosphate derivative agents for remineralization could not provide definitive recommendations on the use of any of the investigated agents because of low certainty of evidence where the included trials suffered from a high or unclear risk of bias, imprecision of the measurements, and indirectness of the outcome measures. In a scoping review, Anil et al. [20] concluded, on the basis of evidence from in vitro, in situ, and in vivo clinical trials, that the efficacy of nHAp dentifrices to remineralize early lesions is limited. The review stressed the need for better studies with consistent outcome measures.

Remineralization of enamel lesions has been attempted with a wide variety of fluoride products. These products often do not provide calcium or phosphate and rely entirely on saliva to provide these essential mineral ions. One theory is that the fluoride absorbed onto the surface of the enamel will selectively attract calcium ions from the saliva followed by phosphate ions, forming a calcium–phosphate–fluoride-like veneer. This veneer will act like fluoroapatite [21]. However, the availability of calcium phosphate in saliva is limited and may not be sufficient for remineralization [22]. Others have attempted to create calcium reservoirs through a calcium rinse, followed by a fluoride rinse to create mineralizing reservoirs [23]. These reservoirs may help prevent demineralization by dissolution but have not been demonstrated to remineralize enamel lesions. Wefel [24] points out that the presence of fluoride in calcium phosphate based remineralization products tends to cause rapid precipitation of calcium–phosphate–fluoride salts resulting in sealing off the tubules of the enamel and not allowing mineral ions to diffuse into the lesion [24].

Mucositis and xerostomia are common sequelae of both chemotherapy radiation therapy of the head and neck region [25]. Mucositis is the inflammation of the soft tissues of the oral cavity where microbial acids cause tissue breakdown. When sufficient saliva is not available to neutralize the acidic environment, the tissues become painfully inflamed. Saliva also deposits calcium phosphate salts in soft tissues, which help buffer the acidic environment. Therefore, one approach to alleviate mucositis is to provide a rinse that acts as a buffer and also deposits calcium phosphate salts on the oral surfaces [25]. 

This study focused on the remineralization of enamel loss at the very earliest stages of enamel caries without the requirement of pH cycling. The earliest stage is the demineralization of the enamel, which occurs while an intact layer is developing on the surface of the enamel. This is called an early enamel lesion, which is the precursor to the white spot lesion (WSL), which is often active where the lesion is progressing or inactive where the lesion progression has halted. The focus on the early enamel lesion here is because the lesion is active and thus more consistent for testing remineralization technologies.

A commercially available product designed to alleviate xerostomia by exogenous application of electrolytes found in natural saliva, including calcium and phosphate, has been shown to be effective in treating mucositis [26] and holds the potential for remineralization of early enamel lesions. The high concentrations of calcium and phosphate salts in SalivaMax^®^, Forward Science LLC, Houston TX USA a supersaturated calcium phosphate rinse (SSCPR), may provide enough of the mineral ions to achieve remineralization of early enamel lesions, but not enough to cause surface precipitation, hindering remineralization. At high pH, calcium phosphate is deposited as amorphous calcium phosphate (ACP), which converts to an apatite-like mineral without pH cycles. This would help avoid acid inflammation of soft tissues and mucositis pain. This study sought to determine the ability of the SSCPR to remineralize artificial WSLs and the pattern of remineralization should it occur. We hypothesized that the calcium phosphate salts provided by the SSCPR rinse would diffuse deep into an enamel lesion and begin the precipitation process, ultimately forming an apatitic mineral resulting in statistically significant remineralization of the WSL. If successful, this product could help the symptoms of both mucositis and xerostomia.

In this study, we utilized a noninvasive method for following changes in dental mineral density. Micro-computed tomography (μCT) is a highly sensitive, non-invasive technology that allows for monitoring the enamel mineral density and lesion depth over time, mapping the pattern of remineralization. μCT was chosen to evaluate changes in early enamel density for its nondestructive nature and its ability to precisely map mineral density within the enamel lesions more effectively than other quantification methods, such as cross-sectional polarized light microscopy or microhardness techniques.

## 2. Materials and Methods

The sample size of the 10 experimental samples and 5 control samples was calculated from the power observed in a previous study [27]. In this study, the ability of fluoride solutions (0, 250, 1100, and 5000 ppm NaF) or amorphous calcium phosphate (ACP)-forming solutions to prevent surface loss from daily toothbrush abrasion and daily acid exposure was evaluated. The samples were treated for 5d through a cyclic procedure of remineralization treatment (4 h), toothbrushing (10 strokes), 2% lactic acid challenge (0.5 h), and saliva-like solution exposure (19.5 h), which resulted in considerable removal of the demineralized surface. The experiment used polarized light to assess remineralization in the sample cross-sections. The mean ± sd of the ACP-forming group was 184 ± 85%, indicating lesion remineralization and buildup of minerals on the surface of the enamel. By comparison, the 5000 ppm NaF treatment resulted in a 64 ± 13% remineralization and no mineral buildup. The pooled standard deviation was 23.2%, and the calculated power from this experiment was 99.7% at an alpha of 0.05. From these results and an assumed difference of 50% between the experimental and the control samples and a pooled standard deviation of 23% (Cohen’s d effect size of 2.16), a sample size of 5/group is calculated to achieve an 80% power at an alpha of 0.05. Ten experimental samples were used to enhance the experimental power of these experiments.

Fifteen caries-free human premolars were sliced to remove the cusps and the root to yield tooth discs 5 mm thick. Three sample columns were formed by grouping five discs per column, which were embedded in X-ray transparent epoxy such that the buccal side of each disc was on the same side. Due to the unknown history of fluoride exposure of extracted human teeth samples, the fluoride content of the outer layer of the samples will vary as well. The outer layer was removed to expose fresh enamel for the procedures and reduce the variation between teeth. The buccal side of each sample column was sanded with 600 grit paper to remove the epoxy on the fluoride-rich outer layer and expose flat fresh enamel surfaces, leaving the other surfaces coated with epoxy. The samples thus have a flat window of exposed enamel with the rest of the surfaces protected with epoxy. A small amount of X-ray-dense dental composite was embedded in the epoxy at the edge of each tooth to serve as a landmark for aligning micro-CT (μCT) images collected over time. Each sample column was attached to a plastic rod for handling. The sample columns were soaked in a 3% high molecular weight carboxymethylcellulose gel containing 1% lactic acid titrated with potassium hydroxide to pH 3.6 for 48 h to stimulate the formation of WSLs. Following enamel lesion formation, the treatment group of the sample columns (two columns, 10 teeth) was suspended four times daily for two minutes each time in stirred solutions of SSCPR (1 packet in 35 mL dH_2_O). The control of one sample column (5 teeth) was suspended in dH_2_O four times daily for two minutes each time. The SSCPR was prepared immediately prior to sample exposures following the manufacturer’s instructions. The SSCPR in this study was SalivaMAX (Forward Science, Houston, TX, USA), a product cleared by the FDA for the relief of oral mucositis and xerostomia. It is composed of calcium salts, phosphate salts, bicarbonate, and chloride. During the interim periods, all sample columns were suspended in freshly made artificial saliva-like solution. 

The artificial saliva-like solution used in the interim periods was based on Fusayama artificial saliva (described by Olssom et al. [28]). The Fusayama saliva-like solution simulates the inorganic content of human saliva composed of NaCl (6.8 mmol/L), KCl (5.4 mmol/L), CaCl_2_•2H_2_O (2.7 mmol/L), NaH_2_PO_4_•H_2_O (5.0 mmol/L), Na_2_S•9H_2_O (21 μmol/L), and Urea (16.7 mmol/L) [29] titrated to pH 7.0 with NaOH. This recipe is based on an average composition of human saliva (without proteins), while whole saliva has a variable composition that changes as the CO_2_ partial pressure changes. This artificial saliva is supersaturated with respect to human dental enamel. The fresh artificial saliva-like solution was made daily because the precipitation of calcium phosphate salts occurs slowly over time, reducing the degree of saturation of the saliva-like solution such that calcium phosphate salts do not precipitate. 

All samples were photographed and scanned with μCT before demineralization (*t* = −2d), after enamel lesion formation (*t* = 0), and at *t* = 2.5d, 21d, and 35d of remineralization. The μCT images were generated with a Scanco μCT-90 (Scanco Medical AG, Switzerland), the scanning parameters were 70 kV, 114 μA, and the integration time was 300 μs, with a voxel size of 4.4 μm^3^ and thus a pixel area (for a 2D image) of 2.66 μm^2^, and the minimum resolution and pixel width was 1.63 μm. Scout X-ray scans were taken of the sample columns in the μCT holder to assure that the columns were positioned vertically, without tipping in the sample holder. This was important so that the images taken over time could be compared without distortions. The mineral density was calibrated using the Scanco hydroxyapatite calibration phantoms as described by Nazarian et al. [30] to maintain constant density measurements over time. This enables the scans from one evaluation to be compared to scans taken at another time. Mineral density profiles were determined for sound enamel and the lesion body.

ImageJ software [31] was used to analyze the μCT images for mineral density changes over time. Because μCT images are similar to images generated via contact X-ray microradiography, which is the current gold standard for measuring mineral densities of partially demineralized tooth sections [32], the same methods for evaluating the mineral density were used [33]. Only one slice is necessary for this evaluation. The lesion depth was the maximum depth measured in each slice (image). The pixel density values of each tooth were normalized to the percent density value relative to sound enamel defined as 100% density and the black (outside of the tooth) set at 0% density. The composite markers were utilized to achieve registration of the images of the same tooth at various time points. TMR radiographs are not linear at the extremes of the grey scales ([33] Schmuck 2010) and neither are μCT grey scales. The typical linear zone is between 20% and 80% relative mineral density. Should the outside edge of the tooth or the boundary of the lesion be slightly sloped, it is not possible to know where the true edge of the tooth or the boundary of the lesion are located. Therefore, we define the threshold between mineralized–demineralized enamel to the density of 80% of the sound enamel and term this as the Boundary. The depth of the lesion was defined as the distance from the original (pre-demineralization) surface to the Boundary. The Boundary region between 80% and 100% relative mineral density will have a depth of 5 to 10 μm, which will vary greatly depending on the chemistry of the enamel under attack. The percent remineralization was calculated from the Boundary over time relative to the original lesion depth after demineralization for each tooth sample. For example, if the tooth were to fully remineralize (i.e., should the Boundary return to the original pre-demineralized surface), the percent remineralization would be 100%.

The mean and standard deviation were calculated for the percent remineralization at each assessment time for the experimental and control groups. Student’s t-test was used to compare the two means for statistical significance at each time. To evaluate the changes in percent remineralization over time, a 2-way ANOVA was used. A *p*-value of ≤ 0.05 for all tests was considered to be statistically significant.

## 3. Results

**Figure 2:**
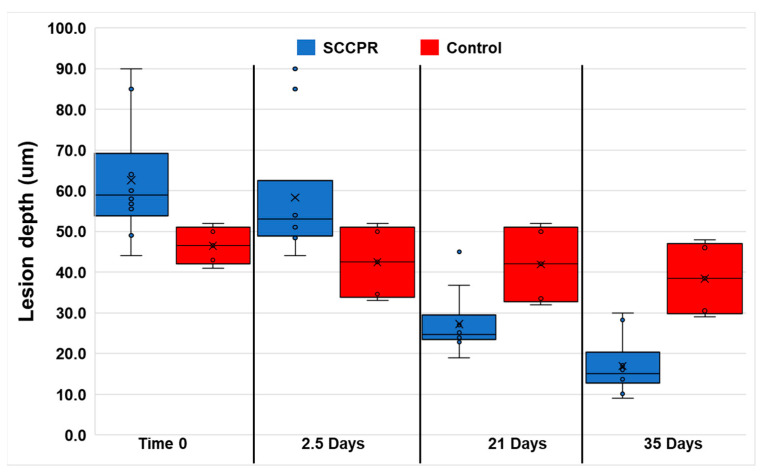
Box and whisker plot showing all the data points for the measurements of lesion depth (μm) during the experiments. The “X” in each box is the average, and the data points are shown along with the standard deviation for each group. The experimental samples were significantly remineralized (*p* < 0.0001), while the control samples did not significantly remineralize (*p* > 0.05).

The mean ± standard deviation of the initial lesion depth of the samples was 57.3 ± 14.3 μm with a range of 41.0 to 90.0 μm. Figure 2 shows a box and whisker plot of the lesion depth (μm) at the starting time (0 days) and 2.5, 21, and 35 days for each of the experimental and control samples. The lesion depth became significantly smaller (*p* < 0.0001) from day 2.5 of the experiments to the end at 35 days. However, the control group did not significantly remineralize over this time (*p* > 0.05).

**Figure 3:**
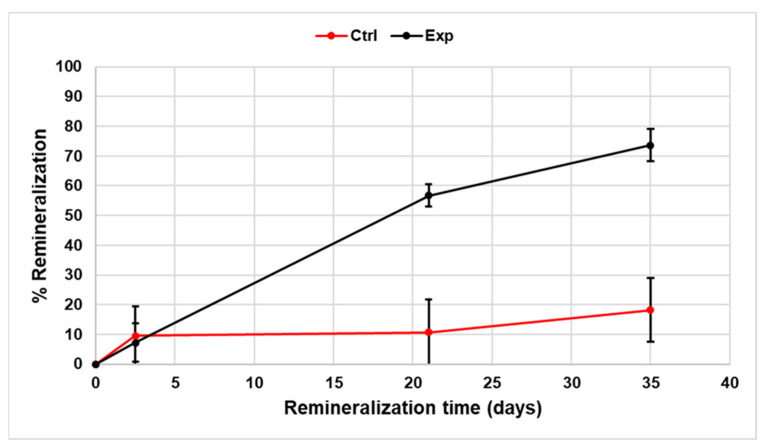
This is a figure that shows the relative percent lesion remineralization over time where the Boundary advances toward the original pre-demineralized surface. After 21 days of exposure to the SSCPR rinse, the % remineralization of the experimental teeth was significantly greater than the controls. The small amount of remineralization of the control samples came from the calcium phosphate-containing saliva-like solution.

To facilitate easier comparisons between the experimental and control groups, the relative percent remineralization was calculated from the net change in lesion depth at the lesion boundary from Time 0 (the start of remineralization treatments) for each sample. Table 1 and Figure 3 show the mean and standard deviation of the relative percent remineralization for the experimental and control samples. The difference between the relative percent remineralization for the experimental versus the control samples was statistically significant at 21 and 35 days (*p* < 0.0001, *t*-test, power = 100%). Two-way ANOVA found that treatment (experimental rinse) and time and their interaction were statistically significant (*p* < 0.0001, power = 100%). There was no significant difference between the two experimental samples (*p* = 0.5897). 

**Figure 4:**
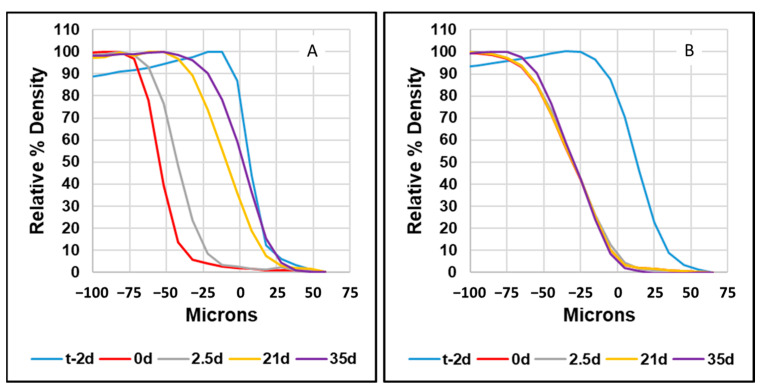
This is a figure that shows the relative densities of a single example from the experimental group (**A**) and the control group (**B**) over time. The initial edge of the enamel (Boundary) is defined as 80% mineral density of the enamel prior to demineralization, which is set at 0 μm in the graphs. Figure 4A and Figure 5 show that the mineral density increased from the deepest part of the lesion toward the surface over time rather than developing a mineral-dense layer at the surface.

**Figure 5:**
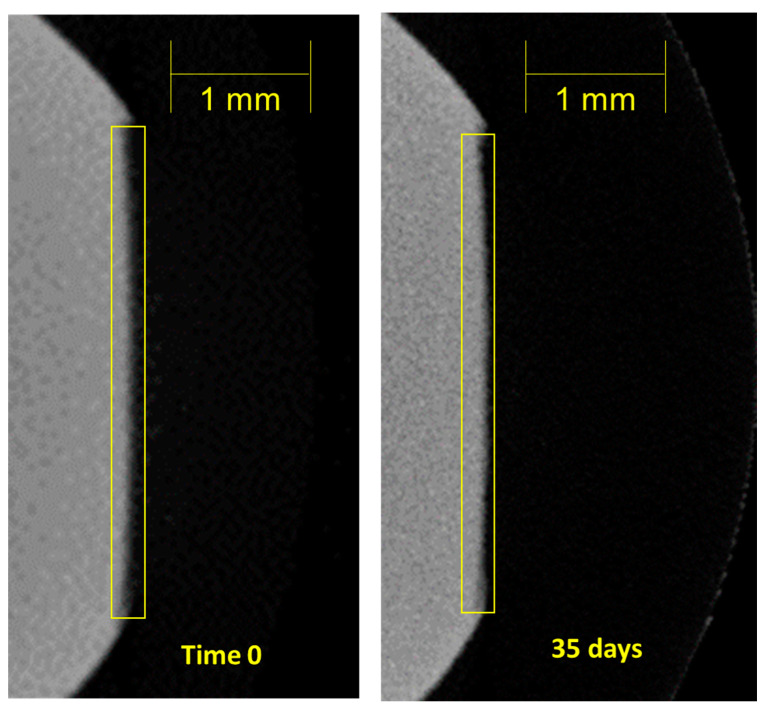
This is a figure that shows the pattern of remineralization of a sample. At Time 0, the tooth surface outlined by the box is mostly missing compared to the same tooth surface at 35 days of remineralization. The SSCPR rinse has deposited significant amounts of calcium and phosphate in the lesion to restore the lesion.

Figure 4 shows the percentage densities, relative to sound enamel, of a single example from the experimental group (A) and the control group (B) over time. The initial edge of the enamel (lesion boundary) is defined as 80% mineral density of the enamel prior to demineralization, which is set at 0 μm in the graphs. The red curve is the Boundary after demineralization and the next curves are the Boundary at 2.5, 21, and 35 days of remineralization. Figure 4A and Figure 5 show that the mineral density increased from the lesion boundary (deepest part of the lesion) toward the surface over time rather than developing a mineral-dense layer at the surface.

Figure 5 shows the μCT density of the enamel lesion for a sample treated with SSCPR, indicating a mineral deposition pattern starting at the deepest part of the lesion moving toward the original surface. Thus, at Time 0, the tooth surface outlined by the box is mostly missing compared to the same tooth surface at 35 days of remineralization.

## 4. Discussion

The results of these experiments support our hypothesis that the calcium phosphate salts provided by the SalivaMax™ rinse would diffuse deep into an enamel lesion and begin the precipitation process, ultimately forming a mineral-dense apatitic mineral resulting in statistically significant remineralization of the lesion. The use of μCT to measure mineral density across a lesion is non-destructive and thus allows for multiple measurements of samples over time. Therefore, in this study, changes in mineral density were able to be monitored for the amount and location of remineralization, as shown in Figure 4.

The process of remineralization of a WSL (and the early enamel lesion) is nuanced and has several requirements. The approach must be carefully balanced to avoid too much precipitating calcium phosphate onto a lesion to avoid plugging the tubules and blocking the uptake of mineral ions into the lesion [24,34]. A brief review of the thermodynamics and kinetics associated with the dissolution and precipitation of calcium phosphate phases helps explain the benefits of the ACP-based strategy to achieve lesion remineralization. Typically, the precipitation of crystalline dicalcium phosphate dihydrate (DCPD) is fast at high calcium and phosphate concentrations below pH 6 (DCPD), creating large crystals that cover the tubules [35]. However, under high pH conditions, the precipitation of amorphous calcium phosphate (ACP) predominates, which may take several minutes [36]. It can be observed that when mixing a concentrated solution of calcium chloride with a concentrated solution of potassium diphosphate at high pH in a test tube, there is rapid ion association forming a gel-like solution, which in 2 min begins the rapid precipitation of ACP. Taking advantage of the time delay prior to precipitation is key to successfully remineralizing a lesion. Tung and Eichmiller [36] provide an elegant description of the thermodynamics associated with ACP and how it spontaneously hydrolyzes from ACP to HAp at neutral pH. This is because ACP does not have a large crystalline structure, which would stabilize a crystal, and thus has a much higher apparent solubility compared to HAp, nHAp, and beta-tricalcium phosphate (TCP) [37]. This higher solubility under normal oral conditions leads to the formation of HAp deep within the lesion [38].

It has been reported that it is possible to penetrate into the lesion with pre-formed calcium phosphate crystals (nHAp, HAp, TCP) and complexes of protein-stabilized ACP (CPP-ACP) [7]. However, none of these phases are able to dissolve at the normal pH range of saliva that is above pH 7 [9,12,39]. Thus, a low pH cycle is needed to release ionic calcium and phosphate to be available for interaction with the existing crystal structure or reprecipitate into another calcium phosphate phase within the lesion [7,24]. The oral environment goes through acidic cycles that are typically below pH 5, and any embedded calcium salt/complex will be dissolved prior to enamel crystals, preventing demineralization. It is possible that sufficiently embedded calcium salt/complexes will be present to release sufficient calcium and phosphate ions to result in remineralization after the acid attack has passed [7], but they will be depleted until the next application of the calcium phosphate salt/complex.

The SSCPR product is an ACP-forming rinse that provides calcium salts and phosphate salts which, when mixed into water, produce high concentrations of calcium ions and phosphate ions. These rapidly move down their diffusion gradients into the lesion where the formation and precipitation of ACP occurs. The ACP-forming rinse can be used multiple times a day. The ACP formed does not require an intermittent acidic pH cycle to hydrolyze to HAp in the lesion. This study has provided the fundamental step of showing that the SSCPR system is able to deliver free mineral ions to the demineralized enamel at a neutral pH to achieve remineralization without the acidic pH cycles. Limitations of this study include that it was in vitro, where many confounding variables that would be encountered in the oral environment were controlled. The fluoride-rich outer layer of the enamel was removed, and pH cycling was not performed to be able to observe the unique chemistry associated with the ACP-forming rinse and assess the ability of multiple applications of the rinse to remineralize early enamel demineralization. While this study reports in vitro remineralization at the advancing front of an oral rinse, an obvious limitation of this study is that artificially induced enamel lesions were used to establish the potential for remineralization. The results of this study cannot be used to inform clinical decisions; however, the information garnered can be used to design further studies to test the rinse in clinical trials. Further studies are needed using naturally occurring WSLs and clinical studies to determine the efficacy of this rinse to remineralize teeth.

SalivaMax™ rinse is a commercially available supersaturated calcium phosphate rinse used up to five times daily for dry mouth and mucositis. The high concentrations of calcium and phosphate, key minerals in tooth composition, position it as a potential solution for remineralization. This study established that calcium phosphate containing SalivaMax™ was able to cause significant remineralization of artificial WSLs. Notably, remineralization occurs at the advancing front of the lesion (deepest zone), which is the most important place to remineralize [40]. Rinse-based remineralization technology would allow the patient to self-administer the remineralizing solutions. This both reduces the costs associated with remineralization and makes it possible for remineralization therapy and treatments for xerostomia and mucositis all in one rinse, which can be applied on a much more frequent basis.

## 5. Conclusions

A marked increase in remineralization occurred with the use of the SSCPR. Notably, the remineralization of the SSCPR occurred deep within the tooth and progressed toward the surface over time. Further clinical research is needed to demonstrate the ability of the rinse, which provides relief for xerostomia and mucositis, allowing them to remineralize early enamel lesions without the requirement of acidic pH cycling.

## Figures and Tables

**Table 1:** The mean and standard deviation of the relative percent remineralization for the experimental and control samples. The difference between percent remineralization for the experimental versus the control samples was statistically significant at 21 and 35 days (*p* < 0.0001, *t*-test).

	Time 0 (%)	2.5 Days (%)	21 Days (%)	35 Days (%)
**Control (dH_2_O)**	0.0 ± 0.0	9.5 ± 9.8	10.7 ± 11.0	18.2 ± 10.8
**Experimental** **(SalivaMax™ rinse)**	0.0 ± 0.0	7.2 ± 6.4	56.7 ± 3.7	73.7 ± 5.4
***t*-test *p***	-	0.5897	<0.0001	<0.0001

## Data Availability

The data associated with this study are available from the author.
